# Random X-chromosome inactivation dynamics *in vivo* by single-cell RNA sequencing

**DOI:** 10.1186/s12864-016-3466-8

**Published:** 2017-01-17

**Authors:** Menghan Wang, Fangqin Lin, Ke Xing, Li Liu

**Affiliations:** Key Laboratory of Gene Engineering of the Ministry of Education, State Key Laboratory of Biocontrol, School of Life Sciences, Sun Yat-sen University, Guangzhou, People’s Republic of China

**Keywords:** X-chromosome inactivation, Single-cell analysis, High-throughput nucleotide sequencing

## Abstract

**Background:**

Random X-chromosome inactivation (rXCI) is important for the maintenance of normal somatic cell functions in female eutherian mammals. The dynamics of X-chromosome inactivation initiation has been widely studied by assessing embryonic stem cell differentiation in vitro. To investigate the phenomenon in vivo, we applied RNA sequencing to single cells from female embryos obtained from a natural intercrossing of two genetically distant mouse strains. Instead of artificially assigning the parental origin of the inactive X chromosome, the inactive X chromosomes in this study were randomly selected from the natural developmental periods and thus included both paternal and maternal origins.

**Results:**

The rXCI stages of single cells from the same developmental stage showed heterogeneity. The high resolution of the rXCI dynamics was exhibited. The inactivation orders of X chromosomal genes were determined by their functions, expression levels, and locations; generally, the inactivation order did not exhibit a parental origin preference. New escape genes were identified. Ohno’s hypothesis of dosage compensation was refuted by our post-implantation stage data.

**Conclusions:**

We found the inactivation orders of X chromosomal genes were determined by their own properties. Generally, the inactivation order did not exhibit a parental origin preference. It provided insights into the gene silencing dynamics during rXCI in vivo.

**Electronic supplementary material:**

The online version of this article (doi:10.1186/s12864-016-3466-8) contains supplementary material, which is available to authorized users.

## Background

Female eutherian mammals randomly inactivate one of the two X chromosomes to equilibrate expression between the sexes in somatic cells [1], although species vary in their early development [2–4]. The time period for the initiation of random XCI in mice is during post-implantation, which occurs at approximately 5.0–7.5 days post coitum (dpc) [5, 6]. The daughter cells inherit the inactivation pattern after initiation [7].

X chromosome gene silencing has been proposed to be controlled by the X inactivation centre [8, 9]. In mice, a 100–500 kb region contains 5 vital noncoding genes that are involved in the regulation of X chromosome inactivation (Xist, Tsix, Xite, RepA and Jpx) [10]. Xist recruits Polycomb repressive complex 2 (PRC2) to form the Xist-PRC2 complex. To initiate XCI, the Xist-PRC2 complex spreads along the whole inactive X chromosome *in cis* and is blocked from binding the active X chromosome by Tsix. The comprehensive Xist interactome has been unravelled [11–13]. The complex methylates lysine 27 on histone H3, leading to chromatin compaction and other epigenetic modifications [14, 15].

Two recent studies revealed the dynamics of Xist localization during XCI initiation using genetically engineered cell lines. The first study found that Xist initially localized on gene-rich islands and then spread to gene-poor domains [16]. The second study demonstrated that the Xist transfer locations were determined by their spatial proximity to the Xist locus rather than based on specific sequences [17]. Both studies concluded that Xist coated the entire X chromosome during XCI initiation but was first located at sites scattered on the X chromosome instead of uniformly spreading from its transcription site. Another study used allele-specific RNA sequencing to investigate the XCI initiation dynamics in vitro. By differentiating of between embryonic stem cells, these authors traced gene silencing due to skewed inactivation on X chromosome from parent 129/SV-Jae. They found that the genes can be stratified into clusters based on their silencing dynamics and that the early silenced genes had a high frequency of close contact with the Xist transcription site [18]. A study of CpG island methylation dynamics on the inactive X chromosome in vitro also showed that kinetics of genes varied [19].

However, the in vivo pattern and whether there is a bias for the parental origin of allelic expression exists are unknown because the parental origin of the inactive X chromosome is often artificially assigned in in vitro experiments. Most studies on rXCI have been conducted on engineered embryonic stem cell lines with either a pre-decided inactive X (Xi) or only one X chromosome and with the inactivated cells synchronized by inducing differentiation. Although a study discussed whether the in vitro reflected the physiological dynamics in vivo, the result was based on a few genes instead of a genome-wide scale [19]. Moreover, the time of inactivation of the X chromosome varies from hours to days in different cell lines or using different differentiation methods, which is not in agreement with the situation in vivo. Thus, whether or not the process represented a real random process should be evaluated.

To investigate the dynamics of rXCI in vivo, we used single-cell transcriptomes of embryos from a natural intercrossing of two genetically distant mouse strains. To the best of our knowledge, this is the first report to explore rXCI dynamics in vivo.

## Results

### Experimental procedure

Two genetically distant mouse strains (C57BL/6 J and PWK/PhJ; hereafter abbreviated as C57 and PWK, respectively) were intercrossed in the study. We used only the female embryos. rXCI occurs early during the development of the female embryo (at approximately 5.0–7.5 dpc) [5, 6]. To validate the rXCI stages of the crossed progenies, we detected Xist expression by RNA fluorescent in situ hybridization (RNA-FISH). The percentages of cells with Xist clouds at 5.5, 6.5 and 7.5 dpc were 7, 45 and 90%, respectively (Table 1). The Fisher’s exact test and Chi-square test showed significant differences between neighbouring stages, suggesting that it was proper to choose female embryos at 5.5, 6.5, and 7.5 dpc to investigate the rXCI dynamics.

**Table 1:** Numbers of cells with Xist clouds at three developmental stages, as detected by RNA-FISH

Developmental stage	Number of cells with Xist clouds	Number of cells without Xist clouds
5.5 dpc	1	13
6.5 dpc	22	27
7.5 dpc	57	6

The Fisher’s exact test showed significant differences between 5.5 dpc and 6.5 dpc (*p* = 0.01)

The Chi-square test showed significant differences between 6.5 dpc and 7.5 dpc (*p* = 4.66 × 10^−7^)

**Fig. 1:**
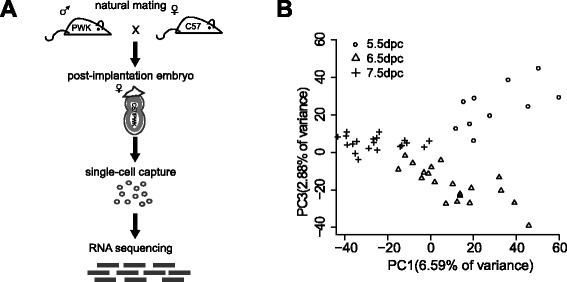
Experimental procedure and PCA of single cell transcriptomes. **a** The work flow of single-cell RNA sequencing from natural mating F1 female embryos. **b** Fifty single-cell transcriptomes projected onto two principal components

After isolation and dissociation of the natural mating F1 female embryos (C57 × PWK), we randomly picked 10, 20, and 20 single cells from embryonic ectoderms collected from 5.5, 6.5 and 7.5 dpc embryos, respectively. To obtain sufficient samples, the 5.5 dpc cells were collected from five embryos, whereas the 6.5 dpc and 7.5 dpc cells were collected from a single embryo per age. The transcriptomes of these cells were sequenced using the Illumina HiSeq 2000 platform. The work flow is shown in Fig. 1a. The embryo genders were determined by PCR prior to single-cell capture and were validated by the mean expression of sex chromosomes using the RNA sequencing data. There were approximately 20.2 million single nucleotide polymorphisms (SNPs) genome-wide and approximately 0.8 million SNPs on the X chromosome between the mouse strains used in this study. These SNPs can be used to provide allele-specific information to distinguish from which parental allele the transcript arose.

The sequencing data were analysed using the SNP-tolerant mapper GSNAP. The following criteria were used to ensure reliable read information: (i) the reads contained only concordant unique mapping and (ii) reads hitting both C57 and PWK were excluded. During these developmental stages, 88% of all expressed genes were observed to contain ≥ 1 allele-specific SNPs (Additional file 1: Table S1 and Additional file 2: Figure S1). Principal component analysis (PCA) was performed on the single-cell transcriptomes (Fig. 1b) and showed that the cells clustered similar to the developmental stages.

### The inactive X chromosome was randomly chosen in vivo

A parameter d was defined to represent the degree of monoallelic expression of each gene. The maternal percentages of gene expression were calculated for both the autosomal and X chromosomal genes in different stages. If the gene is biallelically expressed instead of one allele from a parent being inactivated, d will be 0. If the gene is exclusively expressed from the maternal chromosome, d will be −1. If the gene is exclusively expressed from the paternal chromosome, d will be 1. To minimize the chance that a monoallelically expressed gene was counted due to randomness, the parameter d was only calculated for genes with a total number of allele-specific hits greater than ten. Additionally, the differences in d between autosomal genes and X chromosomal genes were examined by the Wilcoxon rank-sum test for each cell to exclude cells that were not in the process of rXCI (non-rXCI stage). Forty cells showed significant differences in d between autosomes and the X chromosome (Wilcoxon rank-sum test, Additional file 3: Table S2).

By hierarchical cluster analysis (Additional file 4: Figure S2), we identified 4 cells with maternal X-chromosome inactivation (ma-XCI) and 4 cells with paternal X-chromosome inactivation (pa-XCI) at 5.5 dpc. The frequency of ma-XCI was 0.50. Ma-XCI occurred in 10 cells and pa-XCI occurred in 9 cells at 6.5 dpc. Thus, the frequency of ma-XCI was 0.53. Ma-XCI occurred in 4 cells and pa-XCI occurred in 9 cells at 7.5 dpc. Thus, the frequency of ma-XCI was 0.31. The results obtained for 7.5 dpc were a bit unexpected. However, the choice of Xi at 7.5 dpc was statistically in line with a Bernoulli trial (*p* > 0.05). Xi was randomly chosen from the maternal or paternal X chromosome in vivo in this study.

### rXCI showed heterogeneity within a single developmental stage at the single-cell level

**Fig. 2:**
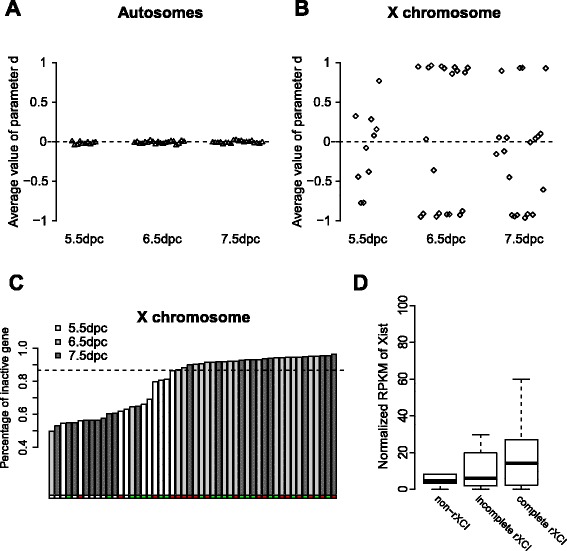
The expression levels of different stages. **a** The average d of autosomal genes for each cell. **b** The average d of X chromosomal genes for each cell. **c** The percentage of inactive genes for fifty cells in an increasing order. The *dotted line* divided the forty cells into incomplete rXCI and complete rXCI. The *red dots* represented cells that were ma-XCI, the *green dots* represented cells that were pa-XCI, and the *white dots* represented cells that were non-rXCI. **d** The normalized RPKM values of Xist for the different rXCI stages

To examine the rXCI stages during the different developmental stages, d values were calculated for both autosomal and X chromosomal genes. The average d of the autosomal genes was close to 0 in all three developmental stages, indicating that both alleles of the autosomal genes were expressed. In contrast, the average d of the X chromosomal genes ranged from −1 to 1 (Fig. 2a, b). Few cells at 5.5 dpc were close to −1 or 1 (d < −0.85 or d > 0.85). This result indicated that 5.5 dpc is an early time for rXCI, which was consistent with the RNA-FISH experiment results. The values were close to −1 and 1 for 90% of the cells at 6.5 dpc and 50% of the cells at 7.5 dpc, indicating that one of the two X chromosomes in these cells was inactivated. The degrees of monoallelic expression at 6.5 and 7.5 dpc were high in the single-cell data. However, there were more cells between −1 and 1 at 7.5 dpc than at 6.5 dpc. Therefore, it was not suitable to divide the rXCI stages by the time due to the heterogeneity within the same developmental stage.

Genes with an absolute d value greater than 0.9 were defined as inactive genes. The percentages of inactive X chromosomal genes in each cell varied during the rXCI process (Fig. 2c). The cells could apparently be divided into two different rXCI stages: incomplete rXCI and complete rXCI. Twelve cells fell into the incomplete rXCI group and 28 cells were in the complete rXCI group by hierarchical cluster analysis (Additional file 5: Figure S3 and Additional file 6: Figure S4). The dividing line was approximately 85%. The heterogeneity of rXCI was not due to differences in the cell cycle distribution (Additional file 3: Table S2).

To confirm the division of rXCI stages, the normalized RPKM of Xist was calculated for the different rXCI stages (Fig. 2d). Xist expression was highest in the complete rXCI cells and lowest in the non-rXCI cells, although the differences among the three stages were not significant (*p* = 0.50, between non-rXCI and incomplete rXCI; *p* = 0.29, between incomplete rXCI and complete rXCI; *p* = 0.06, between non-rXCI and complete rXCI).

### A large proportion of genes were inactivated in order

**Fig. 3:**
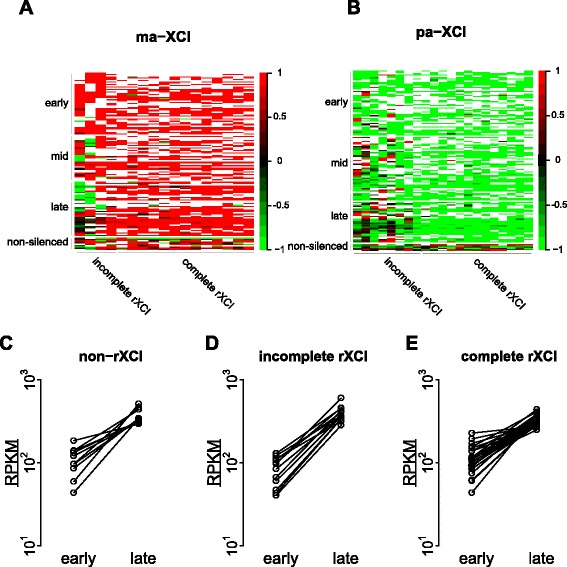
Dynamics of X chromosomal gene silencing. **a** The gene cluster in ma-XCI; the cells were ordered by the proportion of inactive genes. The *colour* represented the d value for each gene in each cell. *White* represented a missing value. **b** The gene cluster in pa-XCI, with similar annotations. **c**, **d**, **e** Expression levels of genes measured by the means of normalized RPKMs (RPKM) in the early and late inactivation sets during the non-rXCI stage, incomplete rXCI stage and complete rXCI stage, respectively

To investigate the dynamics of gene silencing on the X chromosome, the X chromosomal genes were classified into four sets according to their inactivation order (Fig. 3a, b). The definition and details of the gene inactivation sets are provided in the Methods. Genes with more than 10 total hits from the paternal or maternal X chromosome that were expressed in more than half of the incomplete rXCI cells in the groups were included in the following analysis. The genes in the early inactivation set were monoallelically expressed in both the incomplete rXCI cells and the complete rXCI cells. The genes in the mid-inactivation set were monoallelically expressed in the complete rXCI cells but not the incomplete rXCI cells at all the time. The genes in the late inactivation set tended to be monoallelically expressed only in the complete rXCI cells. Finally, the genes in the non-silenced set were biallelically expressed even in the complete rXCI cells; these genes were the candidates of escape genes.

There were a total of 137 and 139 genes in the maternal and paternal inactivation sets, respectively. The 99 genes included in both parental origin inactivation sets were analysed to reveal the differences between ma-XCI and pa-XCI (Additional file 7: Table S3). Interestingly, 38% of the genes were assigned to the same inactivation set in ma-XCI and pa-XCI. This proportion increased to 59% when only genes assigned to the early or late inactivation set were considered, indicating that a large proportion of the genes were inactivated in a certain order, regardless of whether the inactive X chromosome was maternal or paternal. We performed 1000 random samplings for the genes in the early and late inactivation sets to test the tendency of ordered inactivation. The proportion of genes with the same inactivation order in both ma-XCI and pa-XCI as we observed was significantly higher than in the random samplings (*p* < 2.2 × 10^−16^, one-sample Wilcoxon signed rank test). The 9 early inactive genes both in ma-XCI and pa-XCI were not enriched in any Gene Ontology (GO) terms. However, Prps2, Hsd17b10, Siah1b and Naa10 all encoded proteins related to biosynthetic processes. The 15 common late inactive genes were enriched in the function term “RNA binding” in the GO enrichment analysis (fold enrichment: 7.12, *p* = 9.65 × 10^−3^). The ribonucleoprotein binding gene Rbm3 encoded protein has been reported to be the direct Xist partner [11, 12]. These findings suggest that the biallelic expression of these genes in the early rXCI stage may be important because rXCI is regulated by multiple RNAs. The inactivation order of the genes has also been classified in an in vitro study, with most of the overlapping genes in our study in the “intermediate” and “late” sets [18]. One explanation for this result is that the start time in the previous study was earlier than our start time.

The normalized expression levels of the common genes in the early and late inactivation sets were examined. As expected, the expression levels of the late inactivated genes were higher than those of early inactivated genes in the incomplete rXCI cells (Fig. 3d). The pattern continued in the complete rXCI cells (Fig. 3e). We traced the expression levels of the genes in the non-rXCI stage and found that the expression levels of the late inactivated genes were higher from the start (Fig. 3c), suggesting that expression level may be another important factor that contributes to the gene inactivation order.

### The locations of the inactive genes in different inactivation sets in vivo

Four genes in the early inactivation set and 13 genes in the late inactivation set in ma-XCI were assigned to the opposite sets in pa-XCI. Few functional characteristics of these genes showed a link to the sex chromosomes. Whether the inconsistencies of the remaining 17 genes were produced by sampling bias or suggested a different preferential inactivation order between ma-XCI and pa-XCI was unclear. However, it was remarkable that the locations of the two groups of genes on the X chromosome were very close. One group included Pdzd4, Lage3, Fundc2, and Brcc3, and the second included Bex2, Bex4, and Acsl4. One possible explanation is that genes located in close proximity are likely to be inactivated at a similar time.

**Fig. 4:**
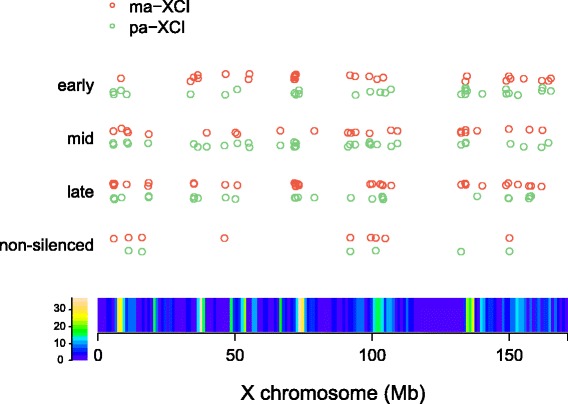
The locations of genes within the inactivation sets on the X chromosome. *Each dot* represented the location of a gene. The gene density on the X chromosome was indicated with a *colour bar*

To investigate the rXCI dynamics, we examined the locations of 99 genes. The gene locations in the same inactivation set highly overlapped in ma-XCI and pa-XCI (Fig. 4). This finding supported the hypothesis that there was no different general preferential inactivation pattern between ma-XCI and pa-XCI. Although the locations of the genes among the three sets showed a spreading trend of expansion from gene-rich to gene-poor regions compared with the early inactivation set and the mid-inactivation set, the difference was not significant (Wilcoxon rank-sum test, Additional file 7: Table S3). rXCI was believed to be initiated in gene-rich regions and it could continue all the time in the gene-rich regions to ensure complete inactivation of the regions. The genes in the early inactivation set were not closer to the X-inactivation centre (XIC) than the genes in the other sets in either ma-XCI or pa-XCI, which was different from the results of a previous in vitro study [18]. Our results supported the hypothesis that Xist coated the entire X chromosome but was first located at sites scattered on the chromosome instead of spreading uniformly from its transcription site during XCI initiation. The distances between X chromosomal genes to XIC ranged from 40 to 50 Mb on average in each inactivation set. The interactions between Xist and the genes in the three sets were calculated using Hi-C data from mouse embryonic stem cells [20]. The data showed no significant differences among the different inactivation sets in both ma-XCI and pa-XCI (Wilcoxon rank-sum test, Additional file 7: Table S3).

### New escape genes found in vivo were homologous to human escape genes

To confirm that the genes from the non-silenced set were escape genes, we filtered genes on the X chromosome as previously described [21] with a modification to increase the stringency of our single-cell data. Briefly, genes that were expressed in at least 10% of the inactive X versus the total active X and inactive X were considered to be escape genes. We identified a total of nine candidate escape genes (Additional file 8: Figure S5). Among them, we identified six previously reported escape genes (Ddx3x, Kdm6a, Eif2s3x, 5530601H04Rik, Kdm5c and Utp14a) [17, 18, 21, 22], and two novel ones, Lpar4 and Rp136a. Xist was also correctly identified as a transcript expressed at a high level in the inactive X.

The six reported escape genes except Utp14a were identified in both ma-XCI and pa-XCI, when analysed separately. Utp14a, Pqbp1, Ogt, and Lpar4 were only identified in ma-XCI, whereas Rp136a was only identified in pa-XCI.

SNPs of these genes were determined by Sanger sequencing of the amplified fragment from cDNA of cells at 7.5dpc to validate the candidate escape genes. Pqbp1, Kdm5c, Rpl36a, 5530601H04Rik and Ddx3x were confirmed to be escape genes (Additional file 9: Table S4). A homologous gene in human of Pqbp1 has previously been reported to be escape gene [23]. In all, we newly identified two escape genes, Rpl36a and Pqbp1.

### Testing dosage compensation during the post-implantation stages

**Fig. 5:**
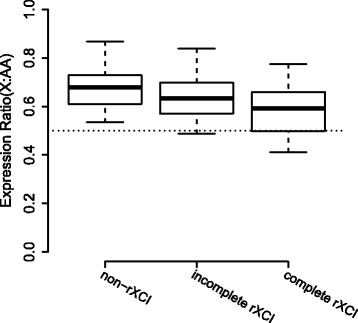
Expression ratio between the X chromosome and autosomes of cells in different rXCI stages

Next, we investigated dosage compensation in mouse post-implantation embryos. Susumu Ohno presumed twofold upregulation of X chromosomal genes to maintain balance between X chromosomal and autosomal gene expression levels in mammalian cells [24]. Although recent studies have not detected widespread upregulation of X chromosomal genes in several organs of adult placental mammals [25, 26], it remains unclear whether there is widespread upregulation during the initiation of rXCI. Genes with one-to-one orthologs in chickens were included. We focused only on highly expressed genes with the same percentages on the X chromosome and autosomes as previously discussed [25, 26]. The embryo underwent rXCI during the post-implantation stages. We discovered significant expression differences between the X chromosome and autosomes in most cells (Additional file 10: Table S5; Wilcoxon rank-sum test). The X:AA ratio decreased with the advance of the rXCI stage and was close to 0.5 in the complete rXCI stage (Fig. 5). These results suggested that the active X chromosome was not up-regulated to balance the X-to-autosome ratio during rXCI.

## Discussion

We exhibited the high resolution dynamics of rXCI in vivo in this study using single-cell RNA sequencing of the crossed progenies of two genetically distant mouse strains. Instead of assigning the parental origin of Xi artificially, in this study Xi was randomly assigned from the natural developmental stages.

Interestingly, the rXCI process in the cells was heterogeneous even within a single developmental stage, indicating that rXCI is not a synchronized step in vivo as shown in the assay of engineered embryonic stem cells in vitro [16–18]. The rXCI of different cell types may occur at different time point as the cells differentiate from embryonic stem cells into the three germ layers. The general trend was consistent with previous reports. The 5.5 dpc cells were in the non-rXCI and incomplete rXCI stages, which was the early time of the rXCI process. The total cell number in the embryonic ectoderm 7.5 dpc was sixty-fold higher than the number of cells at 5.5 dpc and ten-fold higher than the number of cells at 6.5 dpc [27, 28]. The exponential increase in the cell number and the various cell types present at 7.5 dpc may explain the large heterogeneity in the rXCI degree observed at 7.5 dpc. These results are a reminder that distinguishing the rXCI stage by days is not precise and that more cells should be sampled in future studies.

The inactivation order of the X chromosomal genes was determined for both the maternal and paternal Xi. Generally, we did not observe a parental origin bias for the inactivation order. Instead, the inactivation order of genes was associated with their functions. Multiple non-coding RNAs, such as Xist, Xite, and RepA, are recognized to regulate the rXCI process. Genes related to RNA binding, such as Rps4x, Rbm3, and Eif1ax, were in the late inactivation set. It suggested that genes in the late inactivation set may play roles in the early time of rXCI, while the genes in the earlier sets may not. The functions of genes in the early inactivation set also supported this view. In addition to the functions of the X chromosomal genes, their expression levels may also influence the inactivation order.

Although the inactivation order of a number of genes differed between the maternal and parental origins, their locations on the X chromosome were not significantly different. Therefore, we propose that the randomness of rXCI is primarily reflected in the random parental origin choice of Xi; the inactivation order of genes is determined by their functions, expression levels, and locations; within this context, small randomness is allowed for the different parental origin. Because our data were obtained by RNA sequencing, only the transcripts were captured, and other regions on the X chromosome were not included. This limitation may explain why the two-step manner of rXCI initiations previously reported using sequences obtained from RNA antisense purification or capture hybridization analysis of RNA targets with deep sequencing could not be detected [16, 17]. Moreover, the number of captured genes in allele-specific analyses on the X chromosome was around 250 for each single cell in our study, which prevented our ability to deeply analyse the incomplete rXCI stage.

Mice have been reported to possess relatively fewer escape genes than humans. A total of 15% of X chromosomal genes escape X inactivation in humans, whereas 3.3% of X chromosomal genes have been estimated to escape X inactivation in the mouse [21]. Most of these genes are related to brain functions [29]. For instance, Kdm5c plays a vital role in brain development in the mouse, and a mutant *KDM5C* of its homologous gene in human can lead to mental retardation, epilepsy and autistic spectrum disorder [30–32]. In addition to Xist and the reported escape genes, we newly found two escape genes. Among them, Pqbp1 was homologous to human escape genes, indicating that there may be more escape genes in the mouse than expected. There are many ways to test dosage compensation, and most recent studies have been based on whole-transcriptome analysis. The comparison of expression levels between X chromosomal genes and autosomal genes was only meaningful in sex chromosome evolution, that the ancestral X chromosome is represented by present-day autosomes [33]. So we only evaluated one-to-one orthologs. Finally, our data refuted Ohno’s hypothesis of dosage compensation. The X:AA ratio was not upregulated during the post-implantation stages in the mouse.

## Conclusions

Random X-chromosome inactivation is a general process to maintain of normal somatic cell functions in female placental mammals such as human and mouse. It is unclear whether the origin of the inactive X chromosome has any substantive effect. Here, we used mouse embryos obtained from a natural intercrossing of two genetically distant mouse strains. By single-cell RNA sequencing, the differences of gene expression between maternal and paternal inactive X chromosome became comparable. We found the inactivation orders of X chromosomal genes were determined by their own properties. Generally, the inactivation order did not exhibit a parental origin preference. Two new escape genes were identified, one of which was homologous to a human escape gene. Ohno’s hypothesis of dosage compensation was refuted by our mouse post-implantation stage data. These findings are important as it provided insights into the gene silencing dynamics during random X-chromosome inactivation in vivo for the first time.

## Methods

### Mouse mating and embryo collection

All embryos used in the analysis were produced by 8 to 12-week-old C57 mice via natural mating to PWK mice. Vaginal plugs were checked early in the morning the day after mating. The dark midpoint of the day of mating was taken as 0 dpc. Embryos from different post-implantation developmental stages were collected within approximately 2 h around the defined time period.

### Sex determination of embryos and single-cell isolation

The isolated embryos were divided into the extra-embryonic ectoderm and embryonic ectoderm as previously described [28]. The extra-embryonic ectoderm was used to check the gender of each embryo by multiplex PCR using the male-specific Sry gene and the autosomal IL3 gene as previously described [34]. The embryonic ectoderms were placed into the cell culture incubator until the sex of each embryo was determined. Then, the embryonic ectoderm of each female embryo was picked out separately, dissociated in 0.25% trypsin (Invitrogen), collagenase I (0.01 mg/ml; Sigma) and DNaseI (2 U/ml; NEB), and pipetted with a Pasteur pipette (Thermo) until a single-cell suspension was acquired. Prior to passing through a 40 μm cell strainer (BD Falcon), an equal volume of DMEM (Invitrogen) medium was added to the single-cell suspension and the cells were centrifuged at 400 × g for 5 min.

### RNA-FISH

Exon7 of Xist was probed using the Prime-It II Random Primer Labelling Kit (Agilent Technologies) and Cy3-dCTP (GE). The RNA-FISH experiments were performed as previously described [35]. We conducted the Fisher’s exact test and Chi-square test to examine differences in cells with Xist clouds between developmental stages.

### Single-cell capture and transcriptome amplification

The cells were resuspended in an appropriate volume of a mixture with a volume ratio of DMEM supplemented with 10% FBS (Gibco): C1 suspension reagent: C1 wash buffer = 5:3:2 to reach a final concentration of 166,000-250,000 cells/ml. Single-cell capture and mRNA amplification were performed with the C1 Single Cell Auto Prep System (Fluidigm) according to the manufacturer’s recommendations. Briefly, the single-cell suspension was loaded onto a 10–17 micron C1 chip (Fluidigm). After loading the cell suspension, the C1 chip was fast imaged on a microscope. Only a single cell per capture sites was selected as a candidate for RNA sequencing. Subsequently, an mRNA-seq script was run on the C1 System using the SMARTer Ultra Low RNA Kit for Illumina Sequencing (Clontech), including lysis, reverse transcription, PCR and harvest. In summary, we captured 78 single cells from a single 7.5 dpc female embryo and 32 single cells from a single 6.5 dpc female embryo. We captured cells from 5.5 dpc embryos twice (the first time from 3 embryos and the second time from 2 embryos) to obtain a total of 13 single cells.

### Library preparation and RNA sequencing

Single-cell cDNA products were harvested from the C1 chip. The concentration was determined by the Picogreen assay (Invitrogen). A single cell with > 500 pg/μl of cDNA was randomly picked for RNA sequencing. Each sample was diluted to a concentration of 0.13 ng/μl. The cDNA libraries were constructed for each selected sample separately using the Nextera XT DNA Sample Preparation kit (Illumina) following the modified protocol supplied by Fluidigm with minor modifications. Specifically, the 55 °C incubation for 10 min was doubled to 20 min during the tagmentation step. AMPure XP beads (Beckman Coulter) were used to perform a clean-up step. The libraries of the single-cell samples were quantified using the Quant-iT Picogreen dsDNA assay and examined using a high sensitivity DNA chip (Agilent). The single-cell sample libraries were pooled and sequenced using 90 bp paired-end sequencing on an Illumina HiSeq 2000 to an average depth of approximately 15 million aligned reads per cell.

### RNA-sequencing read alignment and SNP-specific read calling

The reads from the HiSeq 2000 with the default filter were aligned to the mm10 genome using GSNAP [36], which is a SNP tolerant mapping software for which we provided intron (refGene.txt from UCSC) information and 20,268,163 SNPs from PWK obtained from the Sanger Institute [37] (Mouse Genomes Project, http://www.sanger.ac.uk/resources/mouse/genomes/). We created the PWK genome by superimposing the PWK SNPs onto the C57 sequence. The reads were aligned to the PWK genome for comparative mapping with intron information and the C57 SNPs. Only concordant uniquely mapping reads were used for the downstream analysis. RPKM values were calculated using rpkmforgenes [38] with refGene.txt as the annotation file. The SNP-specific reads were based on the value of the XV flag, which provides the number of positions that are mismatches against the reference genome but match the alternate genome in the GSNAP sam format output. The reads that hit both C57 and PWK were excluded because the SNPs might be sequencing mistakes. The reads that hit only C57 or PWK were separated into two files as maternal- or paternal-specific reads.

### Principal component analysis

The principal component analysis was based on the expression of all genes. There were ~20,500 autosomal genes and ~1000 X chromosomal genes. RPKM values for each gene in each cell were log_2_-transformed and normalized prior to the analysis. We used PCA to determine the relationships between individual cells.

### Determination of rXCI stages

We evaluated the degree of rXCI of each gene using the parameter d (Eq. 1). The value of d ranged between −1 and 1.

1 $$ d=\frac{0.5-\frac{\mathrm{maternal}\ \mathrm{hits}}{\mathrm{maternal}\ \mathrm{hits}+\mathrm{paternal}\ \mathrm{hits}}}{0.5} $$

when allele-specific hits (the numbers of reads hit maternal- or paternal-specific SNPs) of the gene were equally detected from the maternal and paternal X chromosomes, then the ratio of maternal hits was 0.5 and the value of d was 0. When allele-specific hits were exclusively detected from the maternal X chromosome, then the ratio of maternal hits was 1 and d was −1. If the hits were exclusively from the paternal X chromosome, d was 1. Therefore, we can use the d value to present the degree of monoallelic expression.

Genes with more than 10 hits from either the paternal or maternal X chromosome were included in the following test. We calculated the d value for each gene on the autosomes and X chromosome of each single cell. Prior to the hierarchical cluster analysis, we conducted the Wilcoxon rank-sum test between the d values of genes on the autosomes and X chromosome; only cells with significant differences (*p*_*adj*_ < 0.05) were included in the rXCI process. By considering each single cell as a unit, we conducted hierarchical cluster analysis with multiscale bootstrapping (10,000 replicates) using the d value. Hierarchical cluster analysis was conducted by pvclust R package. The cells in the rXCI stages were divided into ma-XCI and pa-XCI. We applied the hierarchical cluster analysis in a similar manner as discussed above using the absolute d value. The single cells in the cluster with AU *p*-values (Approximately Unbiased *p*-values) > 0.95 were obviously inactivated in most portions of the X chromosomal genes, whereas the other cells seemed to not be inactivated. Therefore, we considered the single cells in the cluster with AU *p*-values > 0.95 to be in the complete rXCI stage, whereas the others were considered to be in the incomplete rXCI stage.

Genes with an absolute d values greater than 0.9 were defined as inactivated genes. We calculated the proportion of inactivated genes for each cell during rXCI and ranked the cells from small to large proportions. One cell was clustered in the complete rXCI but had a smaller proportion of inactivated genes than the two incomplete rXCI cells; therefore, we put this cell into the incomplete rXCI stage.

To eliminate the differences between single cells caused by the cell cycle, we also determined the cell cycle stage for each single cell. The determination of the cell cycle stage was conducted as previously described [39].

### The normalized RPKMs of X chromosomal genes

The RPKM of an X chromosomal gene was divided by a factor (*q*_*i*_) to eliminate differences between each single cell.

2 $$ {q}_i=\frac{\overline{RPK{M}_{autosomal\  genes\  in\  cell\ i}}}{\overline{RPK{M}_{autosomal\  genes\  in\  all\  cell s}}} $$

where $\overline{RPK{M}_{autosomal\  genes\  in\  cell\ i}}$ represents the mean RPKM value of the autosomal genes expressed in cell *i*, and $\overline{RPK{M}_{autosomal\  genes\  in\  all\  cells\ }}$ represents the mean RPKM value of autosomal genes expressed in all cells.

### Random X chromosome inactivation dynamics

We only considered the gene expression patterns in the incomplete rXCI cells when the clustered genes were inactivated in a similar manner in the complete rXCI cells. Genes with more than 10 total hits from the paternal or maternal X chromosome that were expressed in more than half of the incomplete rXCI cells in the groups were included in the following analysis. The clustering of genes was conducted manually because the incomplete rXCI cells were few and most genes had missing values. The genes were divided into four groups: early, mid, late and non-silenced. We defined genes inactivated in more than 60% of the samples as belonging to the early inactivation set, genes inactivated in 40 to 60% of samples as belonging to the mid-inactivation set, and genes inactivated in less than 40% of the samples as belonging to the late inactivation set. The non-silenced cluster was based on the definition of escape genes. The GO enrichment was conducted using AmiGO (http://amigo.geneontology.org/amigo)

The distance to XIC was calculated using the absolute value of the start position of the XIST RNA minus the start position of the other X chromosomal genes. The X chromosome was divided into 171 bins with 1 Mb per bin. The density of genes on the X chromosome was measured by the number of genes in each bin using the start position of the gene. The X chromosomal genes that interacted with Xist were extracted from the Hi-C data for mouse ES cells [20].

### Identification of the escape genes

Because the single-cell data suffered from random losses of RNA, we focused only on genes expressed in at least five cells with at least ten hits (Xi + Xa) for the following analysis. We used the average Xi/(Xi + Xa) ratio of a single cell to represent the cell bulk relative contribution of Xi to the total gene expression similar to the previously described method [21]. The escape genes had an average ratio equal to or greater than 0.1. Genes for which fewer than three single cells passed the criteria were excluded.

To validate the candidate escape genes, primers were designed around SNPs for each gene (Additional file 7: Table S3). cDNA of ten sequenced cells and from another ten cells from 7.5 dpc were used to amplify the specific sequences. After purification of PCR products, Sanger sequencing was performed. For each cell, SNPs of three genes in the early inactivation set (Tceal8, Morf4l2 and Hsd17b10) and Xist were used to determine which X chromosome had been inactivated, which was used to make sure rXCI had happened. The results of the ten sequenced cells were all consistent with previous hierarchical cluster analysis. The genes whose SNPs were heterozygous in more than two cells were considered to be escape genes.

### Dosage compensation of implantation embryos

The analysis included only mouse genes that were one-to-one orthologs with previously described chicken genes [26]. Because the single cell data contained a large proportion of non-expressed and lowly-expressed genes, we selected only highly expressed genes with RPKM values greater than 1 on the X chromosome and the same proportion of genes ranked by RPKM on the autosomes. The X:AA mean expression ratios in each single cell were calculated and divided into three groups according to the rXCI stages.

## Additional files

Additional file 1: Table S1. The alignment results of fifty single-cell RNA sequencing. (XLSX 14 kb)
Additional file 2: Figure S1. Distribution of read hits on SNP sites. A Distribution of read hits on SNP sites in all genes. B Distribution of read hits on SNP sites in X chromosomal genes. SNPs in an increasing order by the number of read hits. (PDF 137 kb)
Additional file 3: Table S2. Summary of stages of each cell by single cell data. The *p*-values of the differences in d values between autosomes and X chromosome for each single cell by the Wilcoxon rank-sum test. The determination of the cell cycle stage for each single cell. (XLSX 15 kb)
Additional file 4: Figure S2. Clustering to determine the parental origin of Xi. Hierarchical cluster analysis with multiscale bootstrapping. Bootstrapping was performed 10,000 times using the d value. The cells in the rXCI stages were divided into ma-XCI and pa-XCI. (PDF 6 kb)
Additional file 5: Figure S3. Dividing the cells into different rXCI stages. Hierarchical cluster analysis with multiscale bootstrapping. Bootstrapping was performed 10,000 times using the absolute d value. Cells in the process of rXCI stages were divided into incomplete rXCI and complete XCI. We considered single cells in the cluster with AU *p*-values > 0.95 to be in the complete rXCI stage, whereas the other cells were considered to be in the incomplete rXCI stage. One cell was clustered into the complete-rXCI but with a smaller proportion of inactivated genes than the two incomplete rXCI cells; therefore, we placed this cell into the incomplete rXCI stage. (PDF 6 kb)
Additional file 6: Figure S4. The percentage of cells in each rXCI stage during different developmental stages. The percentage of cells in each rXCI stage was calculated for each post-implantation stage. (PDF 546 kb)
Additional file 7: Table S3. Summary of genes in inactivation sets. The 99 genes that overlapped in both the paternal and maternal inactivation sets along with descriptions of gene functions. The classified inactivation sets of X-chromosomal genes in ma-XCI and pa-XCI. The *p*-value of locations of genes among the early, mid- and late inactivation sets by the Wilcoxon rank-sum test. The *p*-value of Hi-C of genes among the early, mid- and late inactivation sets by the Wilcoxon rank-sum test. (XLSX 30 kb)
Additional file 8: Figure S5. The Xi ratio of genes on the X chromosome. The red dots represent escape genes and the blue dots represent non-escape genes. The dashed line is 0.1, which is the threshold for escape genes. (PDF 205 kb)
Additional file 9: Table S4. The primers used and the validation of candidate escape genes. (XLSX 11 kb)
Additional file 10: Table S5. The *p*-value of X:AA for each single cell by the Wilcoxon rank-sum test. (XLSX 11 kb)

## Abbreviations

C57: C57BL/6J
GO: Gene Ontology
ma-XCI: Maternal X-chromosome inactivation
pa-XCI: Paternal X-chromosome inactivation
PRC2: Polycomb repressive complex 2
PWK: PWK/PhJ
RNA-FISH: RNA fluorescent in situ hybridization
rXCI: Random X-chromosome inactivation
SNPs: Single nucleotide polymorphisms
Xi: Inactive X
XIC: X-inactivation centre
