# Supplementary figures and images for: Random X-chromosome inactivation dynamics in vivo by single-cell RNA sequencing
Source: BMC Genomics. 2017 Jan 17;18:90. doi: 10.1186/s12864-016-3466-8 (PMC5240438; doi:10.1186/s12864-016-3466-8)

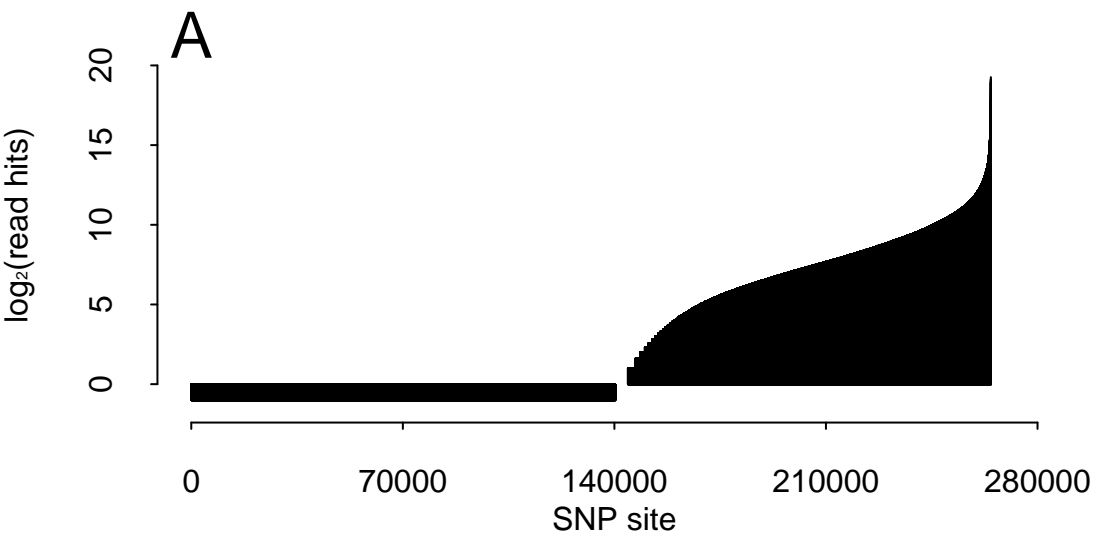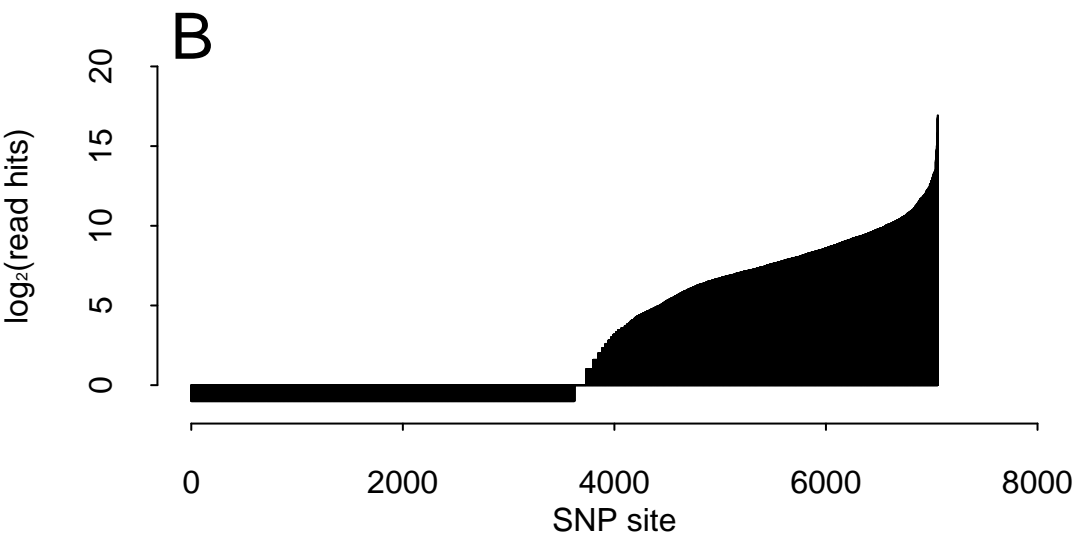

Supplement: Additional file 2: Figure S1. — Distribution of read hits on SNP sites. A Distribution of read hits on SNP sites in all genes. B Distribution of read hits on SNP sites in X chromosomal genes. SNPs in an increasing order by the number of read hits. (PDF 137 kb) [file 12864_2016_3466_MOESM2_ESM.pdf]

Cluster dendrogram with AU/BP values (%)

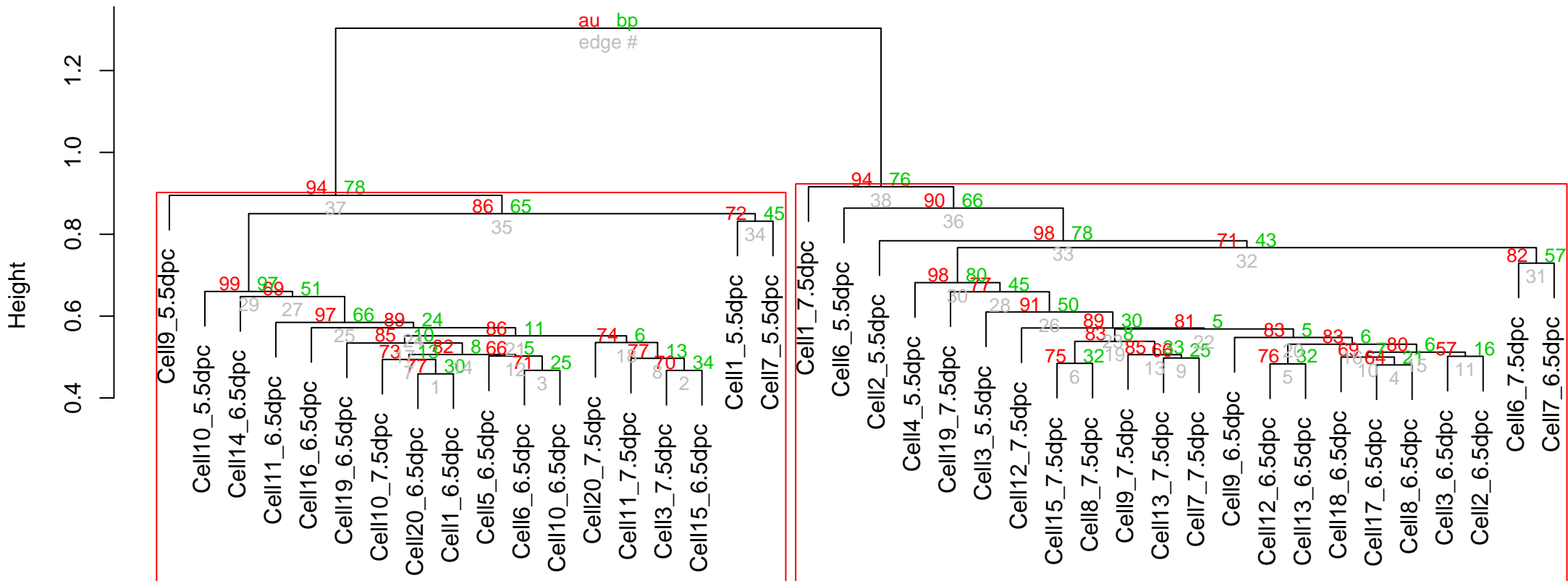

Distance: correlation  
Cluster method: average

Supplement: Additional file 4: Figure S2. — Clustering to determine the parental origin of Xi. Hierarchical cluster analysis with multiscale bootstrapping. Bootstrapping was performed 10,000 times using the d value. The cells in the rXCI stages were divided into ma-XCI and pa-XCI. (PDF 6 kb) [file 12864_2016_3466_MOESM4_ESM.pdf]

Cluster dendrogram with AU/BP values (%)

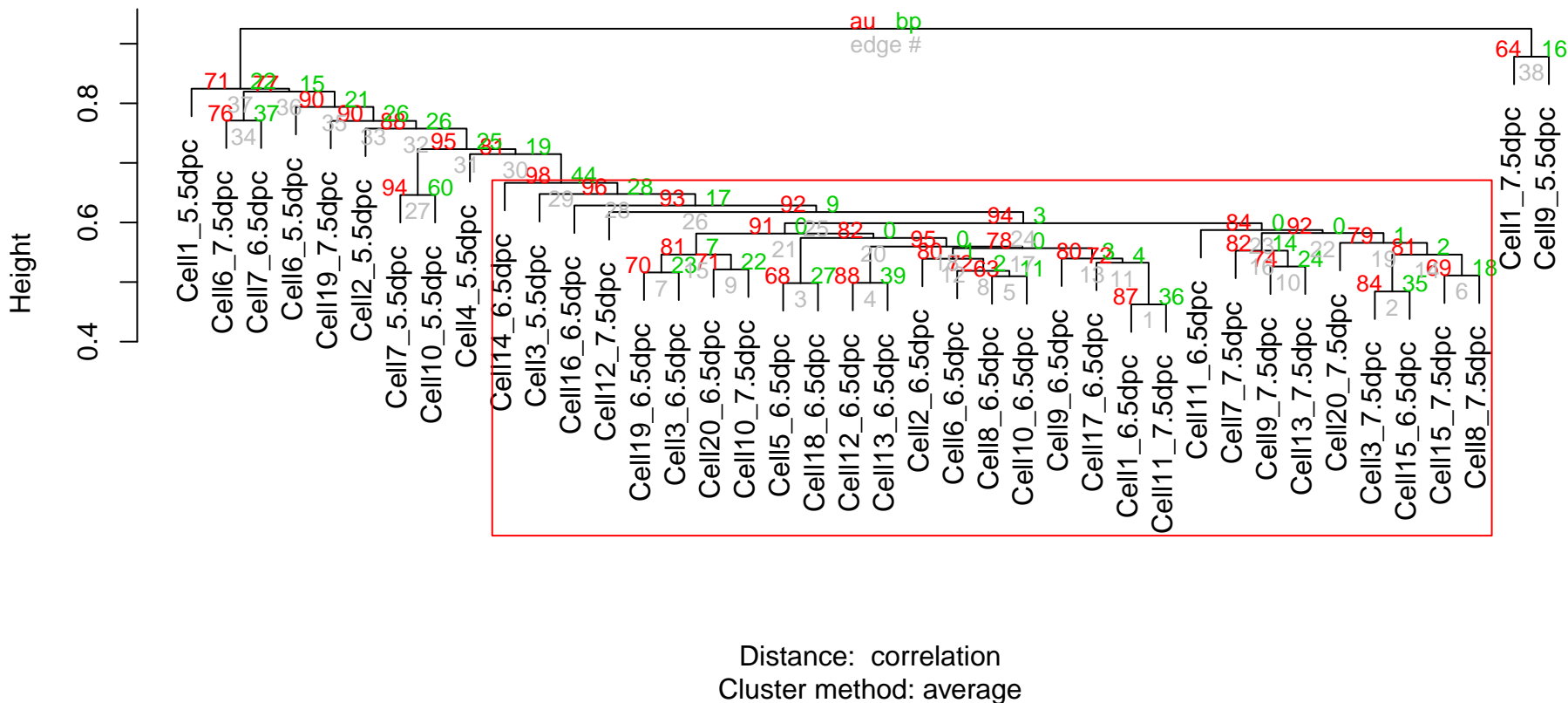

Supplement: Additional file 5: Figure S3. — Dividing the cells into different rXCI stages. Hierarchical cluster analysis with multiscale bootstrapping. Bootstrapping was performed 10,000 times using the absolute d value. Cells in the process of rXCI stages were divided into incomplete rXCI and complete XCI. We considered single cells in the cluster with AU p-values > 0.95 to be in the complete rXCI stage, whereas the other cells were considered to be in the incomplete rXCI stage. One cell was clustered into the complete-rXCI but with a smaller proportion of inactivated genes than the two incomplete rXCI cells; therefore, we placed this cell into the incomplete rXCI stage. (PDF 6 kb) [file 12864_2016_3466_MOESM5_ESM.pdf]

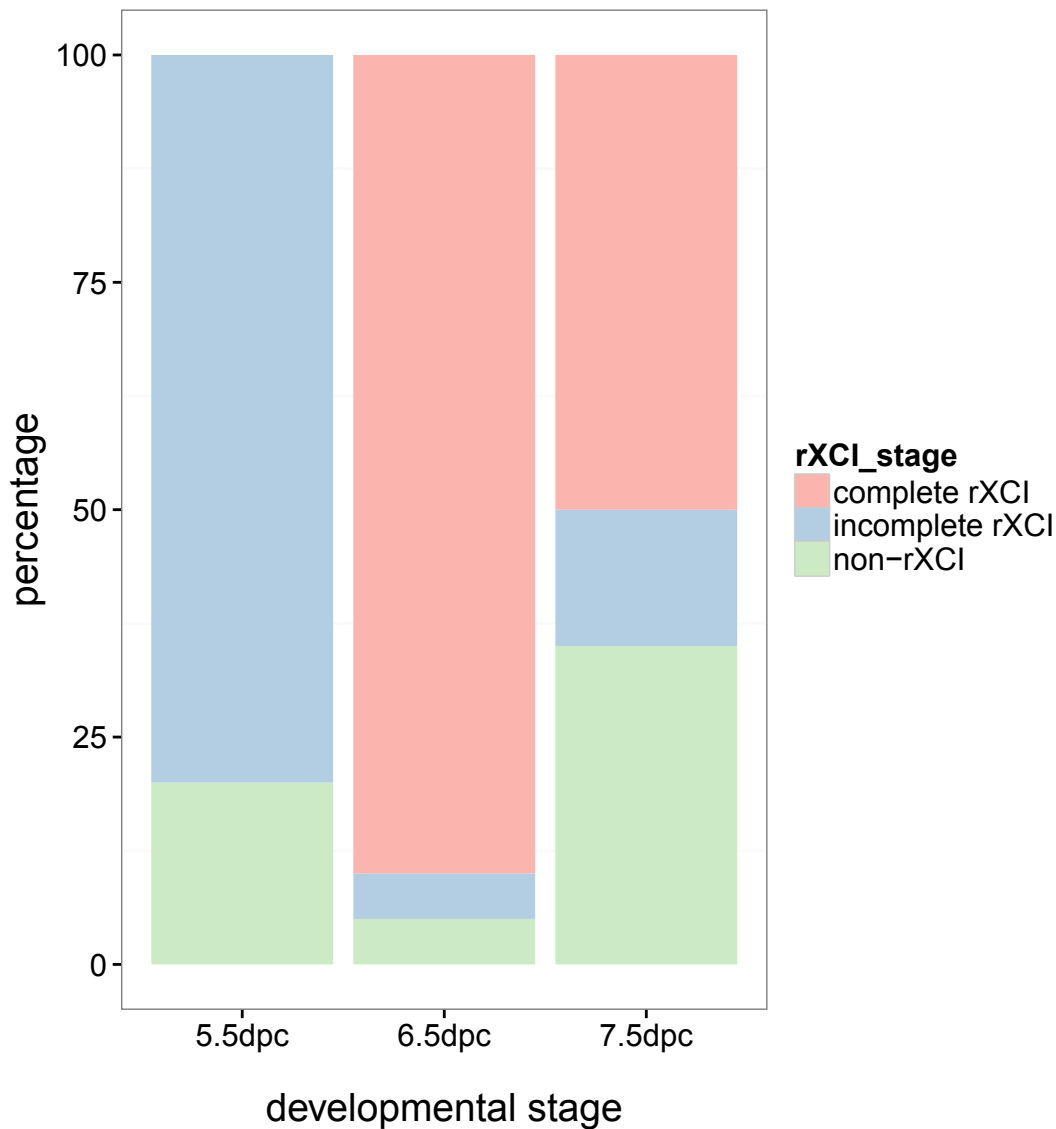

Supplement: Additional file 6: Figure S4. — The percentage of cells in each rXCI stage during different developmental stages. The percentage of cells in each rXCI stage was calculated for each post-implantation stage. (PDF 546 kb) [file 12864_2016_3466_MOESM6_ESM.pdf]

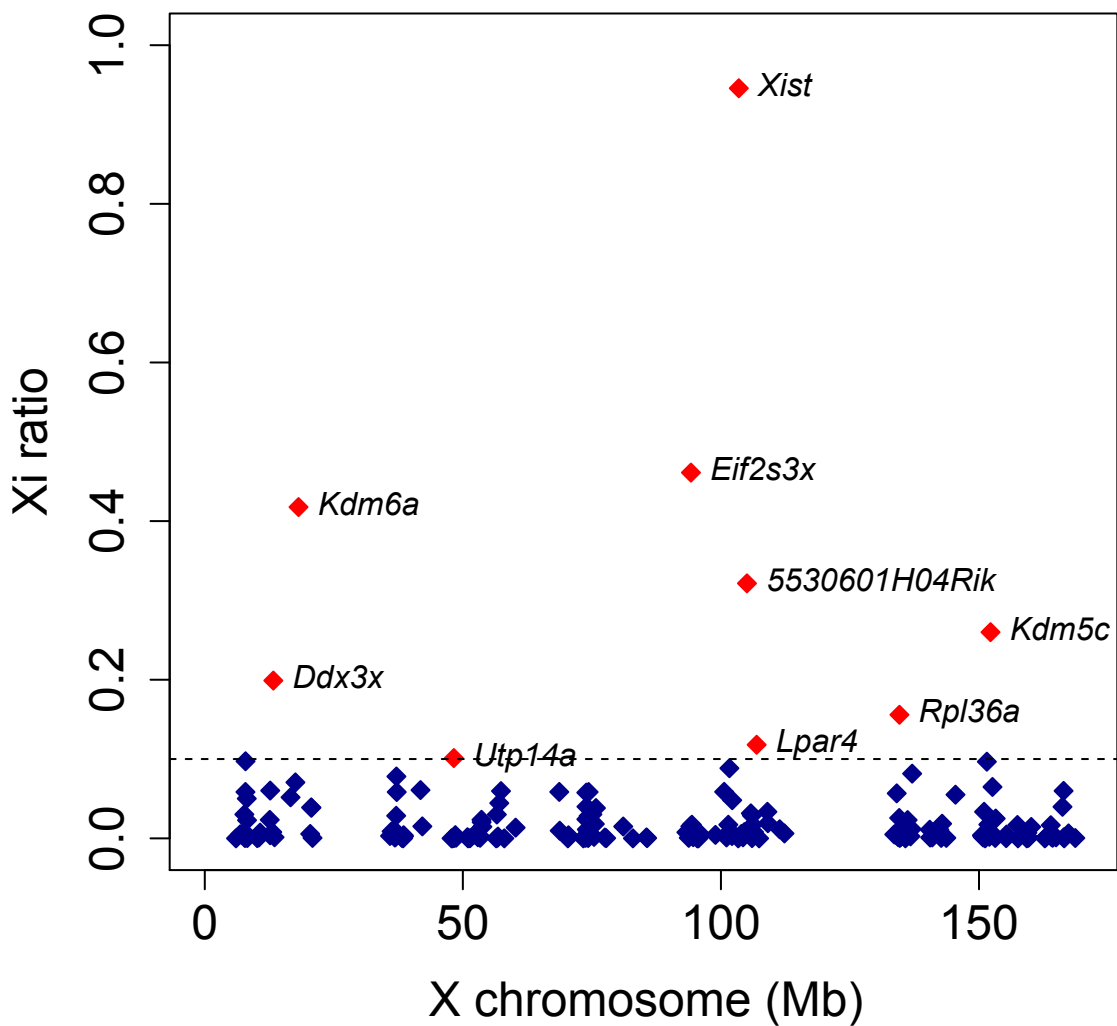

Supplement: Additional file 8: Figure S5. — The Xi ratio of genes on the X chromosome. The red dots represent escape genes and the blue dots represent non-escape genes. The dashed line is 0.1, which is the threshold for escape genes. (PDF 205 kb) [file 12864_2016_3466_MOESM8_ESM.pdf]
